# Cough-Predominant Laryngeal Hypersensitivity Syndrome in a Middle-Aged Woman: A Case Report

**DOI:** 10.7759/cureus.103243

**Published:** 2026-02-08

**Authors:** Muhammad Ammar Iqbal, Rabia Mahmood, Muhammad Armughan Khalid, Mohammad Shaiq Mahmood, Muhammad Shadab Aslam Khan, Umar Khan

**Affiliations:** 1 Respiratory Medicine, University Hospital Limerick, Limerick, IRL; 2 Medicine, William Harvey Hospital, East Kent Hospitals University NHS Foundation Trust (EKHUFT), Ashford, GBR; 3 Medicine, Combined Military Hospital, Lahore, PAK; 4 Internal Medicine, University Hospital Waterford, Waterford, IRL; 5 Respiratory Medicine, University Hospital Kerry, Tralee, IRL

**Keywords:** flexible fiberoptic bronchoscopy, laryngeal hypersensitivity, middle-aged woman, pulmonary function tests (pfts), speech and language

## Abstract

A middle-aged woman presented with a three-month history of sudden-onset persistent dry cough, with no preceding respiratory illness or significant medical history. She was an active smoker with a prior heavy smoking history. Initial investigations, including chest imaging, computed tomography of the neck and thorax, pulmonary function testing, and otolaryngological assessment, were normal. Speech and language therapy evaluation did not reveal any abnormalities of breathing or voice function.

Flexible bronchoscopy demonstrated normal lower airway anatomy but revealed marked laryngeal hypersensitivity, with minimal contact provoking repetitive coughing. In the absence of an alternative pathology, a diagnosis of cough-predominant laryngeal hypersensitivity syndrome was made. Management focused on patient education, hydration, behavioral cough suppression strategies, and measures to reduce laryngeal irritation. This case underscores the importance of considering laryngeal hypersensitivity syndrome in patients with unexplained chronic cough despite normal routine investigations.

## Introduction

Laryngeal hypersensitivity includes a constellation of symptoms as a result of sensory dysfunction of the larynx [1]. Patients can suffer from any of the symptoms like chronic cough, feeling of lump in the throat, burning sensation in the throat or continuous irritation of the throat, tightness of the throat, pain during swallowing food, or sometimes a choking sensation [2]. The cause of laryngeal hypersensitivity syndrome is unknown. However, potential triggers include upper respiratory tract infection with common viruses [3], gastroesophageal reflux disease (GERD), different agents in cigarette smoking, various odors, cold or humid air, chemical irritants in industrial and occupational exposure, side effects of medications, food allergens, or even emotions like anxiety or stress [4]. The laryngeal cough reflex which is a natural defense reflex to protect the airways is exaggerated in laryngeal hypersensitivity syndrome. Persistent coughing over an extended duration may lead to heightened sensitivity of the laryngeal and vocal fold mucosa. As a result, patients can experience a sensation of airway threat or irritation despite the absence of a true obstructive stimulus, creating a perceived urge to cough or clear the throat even when no physical trigger is present [5]. The workup of laryngeal hypersensitivity includes medical history, personal history, occupational history, and assessment from an otolaryngologist, pulmonologist, and speech and language therapist (SALT) [6]. Treatment is multidisciplinary and includes avoidance of potential triggers, occupational therapy, throat care, hydration, management of GERD (if present), special breathing exercises, cough control maneuvers, and gabapentin [7].

## Case presentation

**Figure 1:**
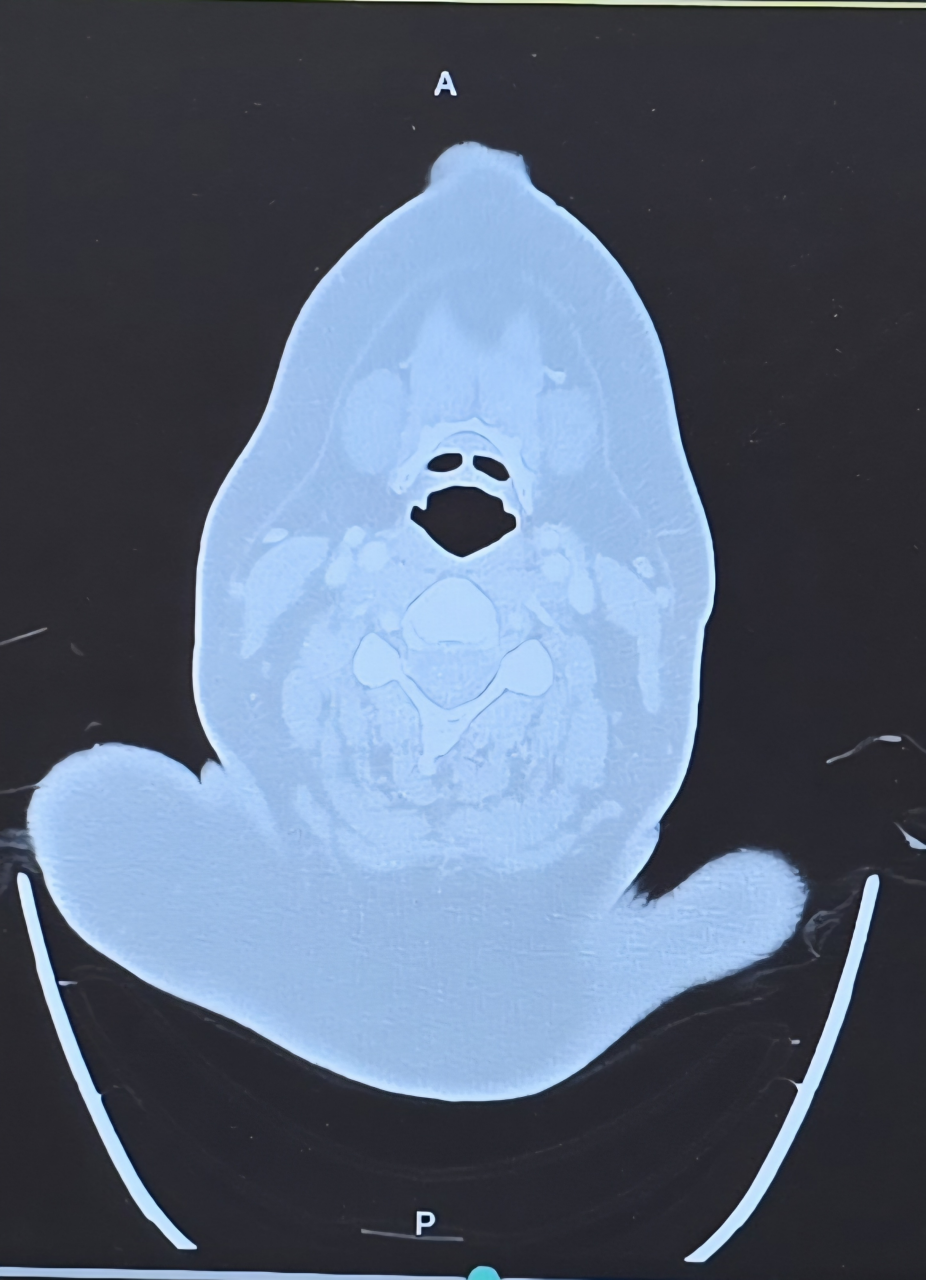
Axial CT image of the neck at the level of the larynx demonstrating normal airway patency and surrounding soft tissue structures, with no evidence of mass lesion, airway narrowing, or structural abnormality to explain chronic cough. CT: computed tomography

**Figure 2:**
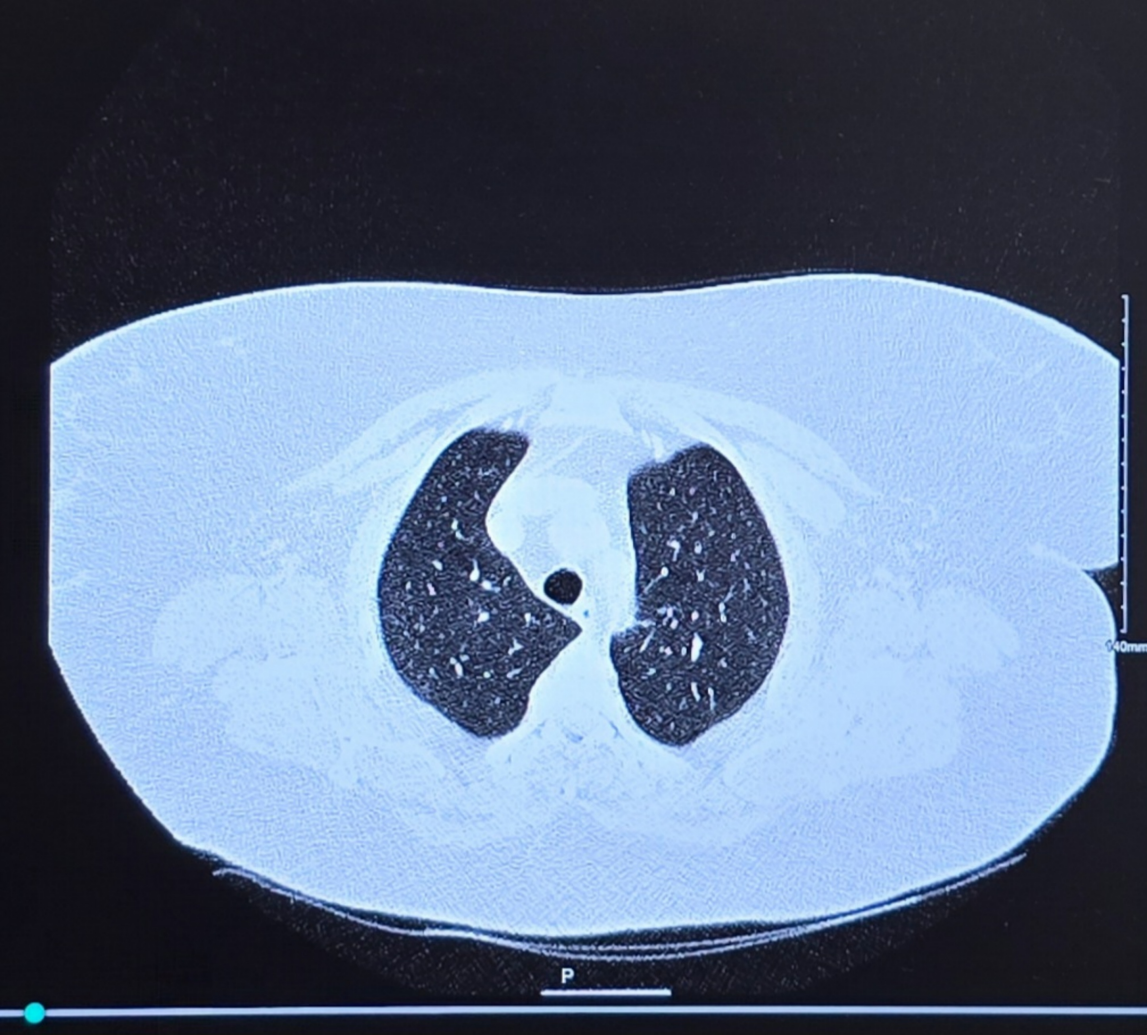
Axial CT image of the thorax at the level of the central airways demonstrating normal lung parenchyma and bronchovascular structures, with no pulmonary abnormality identified to explain chronic cough. CT: computed tomography

**Figure 3:**
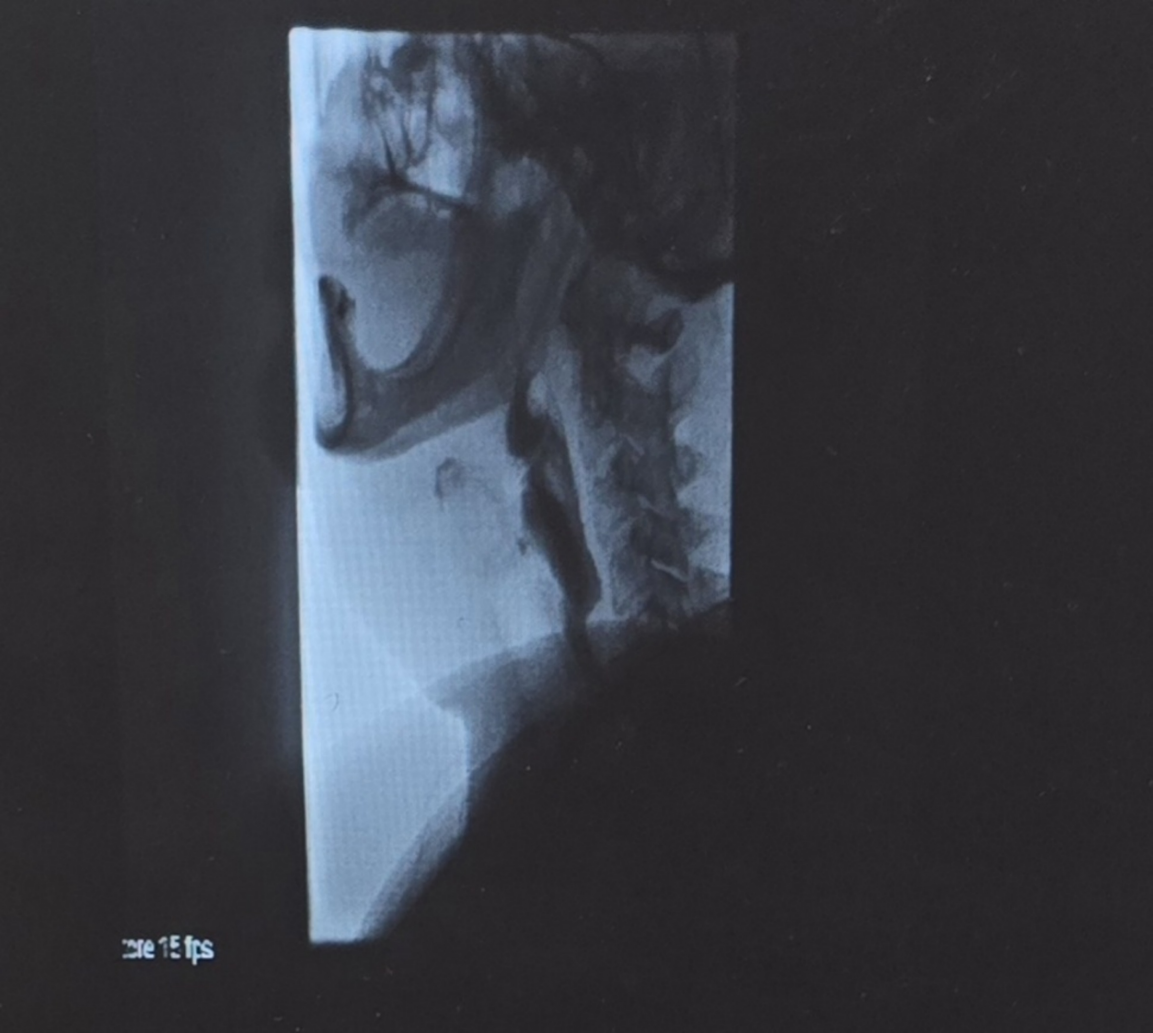
Lateral videofluoroscopic swallow study demonstrating normal oropharyngeal and laryngeal movement with preserved airway protection and no evidence of aspiration or structural abnormality.

A middle-aged woman presented in a tertiary care hospital with symptoms of a chronic dry cough for three months. The past medical, surgical, or psychological history was non-significant apart from a 20-pack-year smoking history. The patient reported something stuck in her throat since her cough started. The cough was extremely disabling leading to disturbance in her sleep. As a result of this cough, she started to get urinary incontinence. Using inhalers or over-the-counter cough syrups didn't help in relieving her symptoms. She was seen by an otolaryngologist who did flexible nasendoscopy and documented bilateral vocal cord movement viewed with no lesion identified. The chest X-ray followed by computed tomography (CT) of the neck and thorax (Figure 1 and Figure 2) was normal. The pulmonary function tests (PFTs) were normal. This was followed by flexible bronchoscopy. The bronchoscopic airway exam was normal; however, the larynx was extremely hypersensitive to touch resulting in bouts of cough despite being given adequate sedation. The patient was then assessed by a SALT who recommended videofluoroscopy. The videofluoroscopy came back as normal (Figure 3).

Based on the workup, she was diagnosed with laryngeal hypersensitivity syndrome. Therapeutic strategies that were considered included providing education on laryngeal physiology and symptom mechanisms, promoting upper-airway hydration, and addressing upper-body muscle tension or excessive laryngeal strain. She was offered psychosocial support as well. She was referred to a smoking cessation programme to quit smoking.

## Discussion

Laryngeal hypersensitivity syndrome is a clinically diverse condition characterized by a heightened sensory response of the larynx, with clinical manifestations that vary among individuals. Common symptoms include chronic cough, a sensation of lump in the throat, throat burning, choking episodes, a sensation of tightness in the neck, and, in some cases, painful swallowing or laryngospasm. Given the nonspecific nature of these symptoms, diagnosis relies on a comprehensive evaluation that includes a detailed medical, occupational, and personal history, along with a multidisciplinary assessment involving otolaryngologists, pulmonologists, SALT, and, when indicated, psychological support services [8].

Management is largely conservative and focuses on reducing symptom burden and minimizing exposure to precipitating factors. Key strategies include smoking cessation, avoidance of known irritants, patient education and reassurance, adequate hydration, and structured breathing and cough suppression techniques. Behavioral and environmental modifications play an important role in improving symptom control and overall quality of life, highlighting the value of a tailored, multidisciplinary approach to treatment [9].

## Conclusions

Laryngeal hypersensitivity syndrome is a rare entity often masked by symptoms of cough. The cause is unknown, but multifactorial etiologies lead to the hyperstimulation of the normal laryngeal cough reflex. The workup is multidisciplinary and includes otolaryngologists, pulmonologists, SALT, and psychotherapists. Patient education is the cornerstone of management. Smoking cessation strategies are a vital part of management. The chronicity of symptoms has long-term social effects and has to be addressed with care and dignity. 
